# Alternate Causes for Pathogenesis of Exfoliation Glaucoma, a Multifactorial Elastotic Disorder: A Literature Review

**DOI:** 10.3390/cimb44030078

**Published:** 2022-03-01

**Authors:** Munmun Chakraborty, Aparna Rao

**Affiliations:** 1 Hyderabad Eye Research Foundation (HERF), L.V. Prasad Eye Institute, Bhubaneswar 751024, Odisha, India; munmun282@gmail.com; 2 School of Biotechnology, KIIT University, Bhubaneswar 751024, Odisha, India

**Keywords:** exfoliation, miRNA, autophagy, mitochondrial dysfunction, blood–aqueous barrier

## Abstract

Exfoliation glaucoma (XFG) is the most recognizable form of secondary open-angle glaucoma associated with a high risk of blindness. This disease is characterized by white flaky granular deposits in the anterior chamber that leads to the elevation of intraocular pressure (IOP) and subsequent glaucomatous optic nerve damage. Conventionally, XFG is known to respond poorly to medical therapy, and surgical intervention is the only management option in most cases. Various genetic and nongenetic factors are known to be linked to the development of XFG. Despite decades of research on the genetic factors in exfoliation syndrome (XFS) by study groups and global consortia involving different ethnic populations, the pathogenesis of XFS and the mechanism of onset of glaucoma still remains an unsolved mystery. The key lies in understanding how the function of a gene (or set of genes) is altered by environmental triggers, along with other molecular events that underlie the key disease attributes, namely, oxidative stress and the disruption of the blood–aqueous barrier (BAB). It remains a challenge to evolve a theory encompassing all factions of molecular events occurring independently or parallelly that determine the disease manifestation (phenotype) or the stage of the disease in the eye (or in any tissue) in exfoliation. Our enhanced understanding of the underlying molecular pathophysiology of XFG, beyond the known genes or polymorphisms involved in the disease, will lead to improved diagnosis and management and the ability to recognize how the environment influences these key events that lead to the disease phenotype or disease progression. This review summarizes the recent observations and discoveries of four key factors that may hold the answers to the non-lysyl oxidase-like 1 (*LOXL1*) mechanisms behind XFG pathogenesis, namely, the epigenetic factor miRNA, disordered autophagy along with the potential involvement of mitochondrial mutations, and a compromised aqueous–blood barrier.

## 1. Introduction

**Figure 1:**
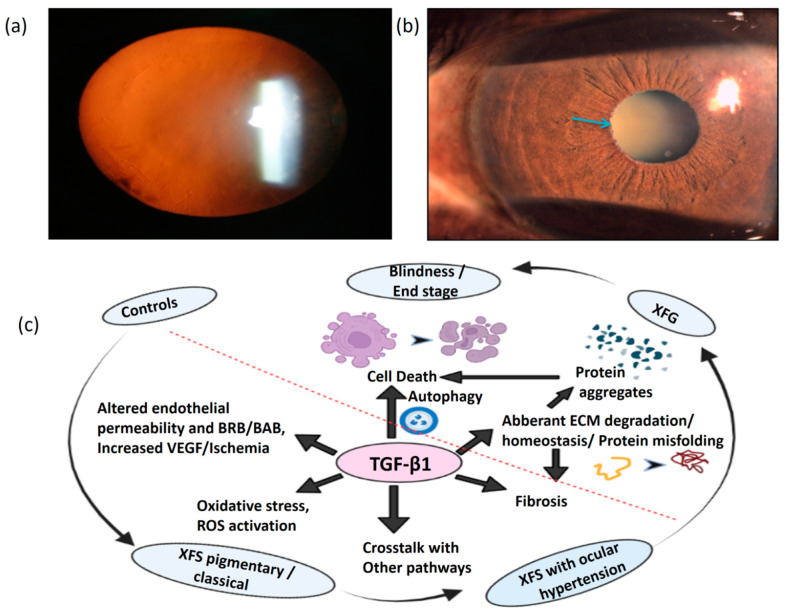
(**a**) Retroillumination showing peripheral granular exfoliative deposits, (**b**) exfoliative deposits (indicated by blue arrow) over the pupil with diffuse stromal iris atrophy, (**c**) diagrammatic depiction of the role of TGF-β1 in various cell processes and the changes that occur as the disease progresses from normal (control) to exfoliation syndrome (XFS) to exfoliation glaucoma (XFG).TGF-β1—transforming growth factor-beta1; BAB—blood–aqueous barrier; BRB—blood–retina barrier; VEGF—vascular endothelial growth factor; ROS—reactive oxygen species; ECM—extracellular matrix. Biorender. Available online: https://biorender.com/ (accessed on 17 February 2022).

Exfoliation glaucoma (XFG) is a severe and progressive type of sight-threatening [1,2] disease, a systemic fibrillopathy, and is one of the most commonly recognizable, clinically unique form of open-angle glaucoma [2,3,4]. Patients with XFG present with more severe optic nerve damage and visual loss at presentation, are refractory to medical treatment, and are associated with a faster rate of progression compared to other forms of primary glaucoma [3,4]. Exfoliation syndrome (XFS) is a unique ocular and systemic disorder characterized by fibrillar exfoliation deposits on ocular structures (Figure 1a,b) and other organs. Though cutting-edge molecular and genetic approaches have provided powerful insights into the genetic and nongenetic factors, the cause and mechanism of the deposit formation and onset of glaucoma in this disease still remains unexplained. A genome-wide association study performed in 2007 by Thorleifsson and coworkers first showed that three single-nucleotide polymorphisms (SNPs) in the lysyl oxidase-like 1 (*LOXL1*) gene (rs1048661, rs1048661, and rs3825942) are strongly associated with a risk of XFG [5]. Other genes implicated in XFG include calcium voltage-gated channel subunit alpha1 A (*CACNA1A*),proteasome maturation protein (*POMP*), transmembrane protein 136 (*TMEM136*), 1-acylglycerol-3-phosphate O-acyltransferase 1 (*AGPAT1*), RNA-binding motif single-stranded interacting protein 3 (*RBMS3*), and semaphorin 6A (*SEMA6A*) [6,7]. Though several groups have identified several key genes responsible for the disease, the genetic etiology is complicated by the frequent occurrence of *LOXL1* SNPs among healthy people, the uncertain role of polymorphisms, and the allele reversal in different ethnic populations [5,6].

Several risk factors have been recognized that predispose people to XFS, including high ambient light, higher temperatures, proximity to the equator, dietary factors such as coffee consumption, and greater exposure to sunlight or outdoor work [8,9]. It is, however, unclear whether these risk factors influence or change the genetic predisposition to XFS or XFG. Further, while oxidative stress is believed to be a key factor in XFS pathogenesis, the precise molecular signaling pathways that are responsible for XFS disease or disease progression to XFG remain unknown.

Transforming growth factor-beta (TGF-β) is a ubiquitous cytokine regulating several key cellular processes, thereby determining cell fate in any tissue. It achieves this by the regulation of downstream matrix metalloproteinases (MMPs) for extracellular matrix (ECM)/collagen homeostasis, cross-talk with other key pathways such as autophagy, fibrosis through Rho-kinases, and gene transcription via canonical and noncanonical pathways (Figure 1c). This is known to be upregulated in eyes with XFS and XFG, though its role in disease pathogenesis in unclear. In our earlier study, we found TGF-β1 to be upregulated in severe glaucoma, though associated with the differential expression of downstream molecules regulating fibrosis or ECM metabolism [10]. Further, this forms an important link for regulating and modulating ECM degradation via MMP9 in XFG, where the activity of the latter is compromised in severe stages despite the continued overexpression of TGF-β1. We also found specific circulatory microRNAs (miRNAs) to be regulating the proteosome clearance–unfolded protein response (UPR) pathway via SMADs, specifically in XFG [10]. TGF-β1 also regulates mitochondrial reactive oxygen species (ROS) production and modulates cell senescence, while oxidative-stress-induced ROS production can cause TGF activation by upstream regulation. Further, stress markers and vascular ischemic events characteristic of XFS may also be regulated by TGF-β1 that regulates vascular endothelial inflammation, which may be the key factor for disrupted blood–aqueous barriers (BAB) in XFS eyes. While genetic factors can cause a predisposition to XFS development or XFG, the role of the above molecular events in precipitating characteristic and well-recognized clinical features in XFS or the onset of glaucoma in these eyes needs an in-depth study.

The purpose of this manuscript is to review the recent and past literature on the pathogenesis and associations of XFG with the less extensively described and studied roles of miRNAs, mitochondrial mutations, altered autophagy, and leaky BABs. An enhanced understanding of the molecular pathophysiology will lead to improved management, diagnosis, and new treatments for glaucoma.

## 2. Methods

We performed a literature search of the PubMed database, identifying all articles associated with XFG and XFS using the following MeSH terms: exfoliation glaucoma, exfoliation syndrome, autophagy, miRNAs, mitochondria, and blood–aqueous barrier. The selection of articles for the review was based on the following criteria: (i) exfoliation glaucoma OR exfoliation syndrome AND autophagy (*n* = 290); (ii) exfoliation glaucoma OR exfoliation syndrome AND mitochondria (*n* = 410); (iii) exfoliation glaucoma OR exfoliation syndrome AND miRNA (*n* = 292); (iv) exfoliation glaucoma OR exfoliation syndrome AND blood–aqueous barrier (*n* = 398). After searching and evaluating, all selected papers were independently examined by the two authors (M.C. and A.R.). Those that did not have validation experiments, included mere hypotheses rather than being evidence-based, or were in conflict between two authors were rejected. Finally, after a thorough review, we narrowed down the focus of this review to 83 articles.

## 3. Potential Role of miRNAs in XFG

MicroRNAs are small, noncoding RNAs, 21–25 nucleotides in length, that regulate gene expression by binding to the 3′-untranslated region (UTR) of specific messenger RNAs (mRNAs) for degradation or translational repression [11,12,13,14,15]. The expression of miRNAs is often typical for a particular tissue or during essential cellular processes [11,12,13,14,15,16]. A single miRNA can modulate the expression of multiple mRNAs that regulate various physiological processes such as hematopoiesis, proliferation, tissue differentiation, cell-type identity maintenance, apoptosis, signal transduction, and organ development by regulating the expression of various genes [16,17,18,19]. They may exist in a stable state within cells or outside cells in biological fluids, including plasma and aqueous humor (AH), vitreous humor, serum, saliva, urine, and tears, and can exist as exosomes or be bound to carrier proteins [20,21]. Previous studies have reported that the expression of miRNAs can be involved in senescence or age-related neurological disorders, diabetes, degenerative arthritis, carcinomas, and cataracts [16,21,22,23,24,25,26,27,28,29,30,31,32,33,34,35,36,37]. In glaucoma, miRNAs can regulate ECM metabolism by regulating TGF-β and can regulate stiffness by the accelerated maturation of ECM proteins, altering the trabecular meshwork (TM) contractile properties, thus accelerating or inhibiting TM cell senescence, or by modulating oxidative- and mechanical-stress-induced damage [20,22,38] in the cell/tissue(Table 1). Alterations in the miRNA levels may indicate pathogenic processes underlying disease or a stage transition of the specific disease. Thus, miRNAs serve as valuable noninvasive biomarkers for various diseases and help to prognosticate the severity of diseases that are caused by the modulation of the specific processes that they regulate [15,27].

The role of miRNAs in glaucoma remains unclear, with several studies reporting miRNAs specifically expressed in the AH or serum in eyes with glaucoma [15,21,23,39,40]. Drewry et al. found three miRNAs (**miR-125b-5p**, **miR-302d-3p**, and **miR-451a**) and five miRNAs (**miR-122-5p**, **miR-3144-3p**, **miR-320a**, **miR-320e**, and **miR-630**) to be significantly differentially expressed in the AH of primary open-angle glaucoma (POAG) and XFG eyes, respectively, compared to controls [41]. Pathway analysis revealed that these miRNAs are involved in potential glaucoma pathways including tight junctions and TGF-β signaling, all of which are known in XFS pathogenesis. Another study, however, found no difference in miRNA expression between the different kinds of primary glaucoma, though **hsa-miR-6722-3p**, **hsa-miR-184**, and **hsa-miR-1260b** were more frequently found in XFG and POAG, respectively [42]. Another study identified higher levels of expression of 20 miRNAs in XFG and POAG patients than in controls, with 6 out of the 20 miRNAs (**miR-637**, **miR-99b-3p**, **miR-4725-3p**, **miR-4724-5p**, **miR-4358**, and **miR-433-3p**) elevated in both plasma and AH [43]. In our earlier study, we found 12 out of 84 miRNAs to be upregulated in XFG. Out of these 12, 3 miRNAs (**hsa-miR-122-5p**, **hsa-miR-124-3p**, and **hsa-miR-424-5p**) were involved in pathways, namely, TGF-β1, fibrosis/ECM, and proteoglycan metabolism with common effectors such as SMAD3/2 [10]. Phenotype comparisons with fibrosis-related miRNA gave similar results, with **hsa-miR-19a-3p** and **hsa-miR-30a-5p** related to proteoglycans being significantly downregulated in ocular hypertension (OHT) compared to XFG. Flaky aggregates are visibly seen deposited on the lenses of XFS/XFG patients. With its monolayer structure and direct exposure to ultraviolet radiation, the lens capsule epithelium is a significant subject for exploring complex elements, including genetics and environmental influences in XFS. Tomczyk-Socha et al. reported the upregulation of **miR-125b** in the lens capsules of XFS patients when compared to controls (cataract), with no significant upregulation in the XFG patients [44].

It is believed that concentrating on the polymorphisms in the miRNA biogenesis pathway and their dynamic interaction with the genes under specific environmental triggers could uncover data for disease anticipation and pharmacogenomics in XFS [44,45,46]. Recently, various studies have reported differential miRNA expression status in the AH and trabecular meshwork, two anatomical structures that are closely related to glaucoma, and linked them to the apoptosis of retinal ganglion cells and IOP [21,38,39]. Fewer studies, however, have reported polymorphisms in miRNA [45,46,47,48]. One study reported rs1057035 polymorphisms in the 3′-UTR of the *DICER* gene to be associated with a decreased risk of XFG, and rs55671916 in the 3′-UTR of the exportin 5 (*XPO5*) gene with an increased risk of XFG [4,47]. As per the miRNASNP database, the polymorphism rs11382316 results in a gain of function of **miRNA-3161** in the genes caveolin-1 (*CAV1),* cytochrome P450 family 1 subfamily B member 1 (*CYP1B1*), and *CACNA1A,* and a loss of function of transforming growth factor-beta receptor 3 (*TGFBR3*). This result seems to further support a previously reported implication of the *CACNA1A* gene in XFS susceptibility [7].

Given the significance of ECM elements to the ordinary physiology of the outpouring pathway, miRNAs that control ECM metabolism could be reasonable targets to impact AH dynamics in XFS eyes. The best-described group of miRNAs that directs ECM digestion is the miR-29 family, including **hsa-miR-29a**, **hsa-miR-29b**, and **hsa-miR-29c**. In a study by Luna et al., the transfection of TM cells with **miR-29b** mimic caused the downregulation of various ECM genes, including collagens and fibronectin, as well as the targeting of genes involved in ECM remodeling (SPARC/osteonectin). Interestingly, persistent oxidative stress induced by incubation at 40% oxygen led to a critical downregulation of miR-29b in TM cell lines that were related to an increased expression of several ECM genes [49]. Strategies to elevate miR-29 expression in TM cells may be advantageous to limit ECM deposition, avert cell loss, and maintain normal levels of AH outflow facility. However, this family has not been studied in targeting TM function in XFG eyes, nor has its role in XFS and XFG eyes been identified in any study. Further, the regulation of miRNA biogenesis and the TGF-β pathway in exfoliation remains unexplored. A detailed study in this direction may give insights into how TGF-β-regulated processes are modulated differentially in disease progression or in different ethnic populations.

In summary, studies so far suggest that miRNAs are key regulatory elements that can control the pathophysiology of the outflow pathway and modulate ECM degradation or gene expression in XFS. These can serve as promising biomarkers for disease progression, treatment responders/nonresponders, and effective diagnosis while offering insights into possible therapeutic strategies for XFG. To understand the maximum capacity of miRNAs as helpful targets in XFG, it will be important to work towards filling these lacunae in the future.

## 4. Autophagy and Mitochondrial Dysfunction and Protein Aggregate Clearance

We recently demonstrated a decreased UPR clearance in XFG compared to earlier forms of the disease associated with increased TGF levels in all disease stages, which suggest the potential regulation of the autophagy pathway and TGF–autophagy cross-talk involved in cell repair and aggregate clearance [50]. Autophagy is an intracellular trafficking system that conveys cytosolic constituents to the lysosome for ensuing degradation, which is crucial for misfolded protein clearance, cell homeostasis by ubiquitin–proteasomal degradation, and cell repair [51,52,53,54,55]. Immunohistochemical and mass spectrometry investigations have uncovered that exfoliative material is a profoundly glycosylated proteinaceous complex that is very impervious to degradation, both inside the body and under experimental conditions [51]. Given the significance of the autophagic clearance of protein aggregates, autophagy-related genes (ATG genes) might be involved in XFG pathology beyond primary glaucoma [53]. Studies have also shown the association of neurodegeneration with mitochondrial dysfunction and abnormal mitophagy [56,57,58]. Impaired mitophagy causes the accumulation of damaged mitochondria that may have a severe impact on acell’s ability to manage oxidative insult and/or ability to clear misfolded proteins, which may be impaired in XFG eyes. A decreased autophagic flux (an indicator of autophagic activity) caused by oxidative stress may be one of the factors that lead to the progressive failure of cellular TM function with age [59,60,61,62,63]. In our earlier study, autophagy genes were abruptly upregulated in severe POAG and primary angle-closure glaucoma (PACG) compared to moderate glaucoma, suggesting the role of autophagy in disease progression [53].

Similar studies on mitochondrial dysfunction and impaired autophagic clearance are sparse in XFS and XFG. A Spanish-based study, evaluating genetic variants in three critical genes of autophagy (ATG16L, ATG2B, and ATG5) in the development of XFS and XFG in a Spanish population, reported no difference in either the genotype distributions or allelic frequencies of the known polymorphisms between XFS/XFG or controls [54]. One study using explant-cultured tenon fibroblasts from XFS and age-matched POAG patients observed 1.38 times bigger fibroblasts that were less organized, and demonstrated a decreased clearance of autophagosomes with abnormal endocytic trafficking in fibroblasts obtained from XFS eyes. This suggested the aberrant autophagic–lysosomal clearance of proteins in XFS eyes [55]. They also reported a higher content of cells with low mitochondrial membrane potential, suggesting impaired mitophagy. Mitochondrial DNA (mtDNA) has a higher prevalence of mutations owing to less-efficient repair systems [55]. The rate of mtDNA mutations is multiple times that of nuclear DNA and is thought to be triggered by ROS formation inside the mitochondria. Although mtDNA encodes only about 1% of the proteins found in mitochondria, it incorporates subunits of oxidative phosphorylation proteins. Izzotti et al. evaluated TM specimens obtained from surgeries of various glaucoma and control patients [59]. They showed that mitochondrial dysfunction induced by oxidative stress is more predominant in POAG and XFG than in other glaucoma forms. XFG specimens had the most elevated levels of mtDNA deletion, oxidative DNA damage, and mitochondrial loss per cell. The mtDNA 4977deletion values seen in XFG were >2 times those recognized in POAG. Studies using advanced whole-mitochondrial sequencing technology have identified 22 mtDNA changes, comprising 7 novel transformations with 36.4% of the mtDNA changes in the complex I mitochondrial gene [60]. One study reported 27 unique novel non-synonymous mtDNA changes in POAG, with 22 changes being potentially pathogenic [61]. Another study reported the absence of any mutation in XFG patients in the various nuclear genes associated with primary glaucoma or in the mitochondrial genome [62,63,64]. Heterozygous optic atrophy type1 (OPA1) is known to influence mitochondrial steadiness and has now been implicated in a few optic neuropathies. It codes for a dynamin-like GTPase protein found in a polymeric structure in the inner mitochondrial membrane that has multiple distinct roles [65,66,67,68], primarily related to maintaining a highly interconnected mitochondrial network. Thomas et al. studied OPA1 mutations in POAG patients and found significantly lower expression levels of OPA1 than in controls. Decreased OPA1 expression, another sign of mitochondrial-actuated apoptosis, may play a part in the advancement of glaucoma in XFS [66]. To test the theory that mitochondrial haplogroups influence the risk of developing glaucoma, a group genotyped 12 single-nucleotide polymorphisms that characterize the European mtDNA haplogroups in controls and two German cohorts with XFG or normal-tension glaucoma. Individuals with haplogroup-U were found to have a lower risk for developing XFG in this study, suggesting mitochondrial dysfunction in XFS pathogenesis [69]. In summary, research related to mitochondrial dysfunction and impaired mitophagy in XFG has been less extensive to date, with very few studies evaluating the role of the environment in triggering the dysregulation of these processes in XFG. There is a compelling need to supplement the existing literature on XFG pathogenesis with functional studies analyzing various populations and different environmental influences on mitochondrial function in XFS/XFG.

## 5. The Blood–Aqueous Barrier in Eyes with Exfoliation Syndrome

The eye, similar to the brain, is an organ endowed with immune privilege, an attribute conferred by complex ocular barrier systems. Two kinds of barriers have been distinguished inside the eye, each described by its unique tissue restriction, immunologic properties, and physiological capacities, namely, the BAB and the blood–retina barrier (BRB) [70,71]. Eyes with XFS frequently show clinical signs of impairment of the BAB [72]. The breakdown of the BAB is confirmed by an elevation in AH proteins. Mice without the *LOXL1* gene, a significant genetic risk factor for XFS and XFG, displayed an increased dispersion of fluorescein at the BAB, indicating the interruption of the ciliary epithelial barrier [73]. Kuchle et al. studied the alteration in the BAB in XFS patients by the histochemical staining of endogenous albumin and reported the impairment of the BAB in XFS that was confined to the iris and, to a lesser extent, may involve the ciliary body [74]. Elevated levels of AH clusterin in XFS, POAG, and XFG cases compared to controls has been reported by several studies [75,76]. Zenkel et al. [77] reasoned that this was due to the disintegration of the BAB and leakage of systemic clusterin into the AH. On the other hand, Doudevski et al. [76] contended that this increase could not be explained by the breakdown of the BAB alone and that local synthesis might therefore play a significant role. Yildirim et al. found that serum interleukine 6 (IL-6) levels were altogether higher in XFS when contrasted with controls, suggesting higher levels of subclinical inflammation and BAB in XFS patients [78]. Kondkar et al. observed that higher plasma tumor necrosis factor alpha (TNF-α) levels might be a marker for the progression of XFS to XFG [79]. What triggers the disruption of BAB in XFS patients is unclear, though some risk factors such as oxidative stress, raised homocysteine, AH nitric oxide (NO), and vascular endothelial growth factor (VEGF) are presumed to play a role. Eraslan et al. revealed high acylated ghrelin/ghrelin proportions in XFG cases and suggested that acylated ghrelin may adversely trigger prostaglandin and NO release in XFG, causing progressive damage [80]. Bleich et al. observed that significantly elevated (twofold) homocysteine levels in the AH and the plasma of XFG patients with aqueous homocysteine was significantly correlated with corresponding plasma levels [81]. VEGF is also known to increase vascular permeability, contributing to disrupted BABs in XFS [82,83]. This may also explain the frequent ischemic systemic associations in XFS patients, including cardiovascular disease, transient ischemic attacks, and vascular occlusive disease. The mean AH and plasma VEGF concentrations and the mean AH NO concentrations were significantly higher in patients with XFG than in controls [82,83,84]. Studies have reported marginally higher mean AH and plasma levels of NO and VEGF in XFG than in patients with XFS, but the differences were not statistically significant. These studies imply a need for further studies on how these factors cause BAB breakdown and precipitate a cascade of protein complex aggregate accumulation over different ocular structures in XFS. It may be worthwhile exploring the role of ambient light and continued TGF-β exposure in the key downstream pathways such as autophagy, the disruption of the BAB, mitochondrial dysfunction, and oxidative stress in the TM cells causing functional damage. Understanding these processes in animal or invitro models would be crucial to identify mechanisms to reverse or dissolve these aggregates and prevent tissue dysfunction in XFG.

## 6. Conclusions

Vast volumes of genetic studies across the globe have failed to explain the complex mechanisms underlying XFS pathogenesis and the onset of glaucoma in these eyes. It is increasingly becoming clear that molecular processes mediated and regulated by environmental triggers hold the key to these perplexing questions. Current knowledge on the role of miRNA, mitochondrial dysfunction/autophagic clearance, or the disrupted BAB does not explain the reason for the differential involvement of some populations or eyes in XFS or even the onset of glaucoma. This review sums up the current ideas and the gaps existing in these areas in XFS research that may give newer insights and therapeutic targets for XFS. The comprehension of the functional consequences and molecular mechanisms of XFG and the targeting of novel therapeutic approaches are the major roads for future research in XFG pathogenesis.

## Figures and Tables

**Table 1:** List of miRNAs identified in exfoliation syndrome (XFS) and exfoliation glaucoma (XFG) and their functions.

miRNA	Accession Number	Function	Reference
hsa-miR-125b	MIMAT0000423	Directly targets P53. TP53 gene is closely associated with lens epithelial cell apoptosis. (validated)	Drewry et al., 2018
hsa-miR-6722-3p	MIMAT0025854	Involved in mitogen-activated protein kinase (MAPK) signaling pathway; forkhead box, class O (FOXO) signaling pathway; and regulation of actin cytoskeleton. (predicted)	Kosior-Jarecka et al., 2021
hsa-miR-184	MIMAT0000454	Involved mainly in response to hypoxia, cardiovascular system development, and apoptosis. Mutations in hsa-miR-184, which were linked with lens/corneal dystrophy and blindness. (predicted)	Kosior-Jarecka et al., 2021
hsa-miR-4634	MIMAT0019691	Is a validated regulator of VAV3, whose deficiency in mice was associated with an ocular phenotype similar to glaucoma, including elevated IOP, selective loss of retinal ganglion cells, and optic nerve head cupping. (validated)	Kosior-Jarecka et al., 2021
hsa-miR-1260b	MIMAT0015041	May play a protective role in the course of glaucomatous neuropathy. Is also an essential regulator of vascular smooth muscle cell proliferation in response to hypoxia. (predicted)	Kosior-Jarecka et al., 2021
miR-122-5p	MIMAT0000421	Controls TGF-β1, protein binding, and ECM-related processes. Has been shown to regulate opteneuin pathway. (validated)	Rao et al., 2020; Drewryet al., 2018
hsa-miR-124-3p	MIMAT0000422	Controls TGF-β1, protein binding, and ECM-related processes. (predicted)	Rao et al., 2020
hsa-miR-424-5p	MIMAAT0001341	Is a tumor-suppressive miRNA. It regulates proliferation and invasion. It can also inhibit cell migration and epithelial–mesenchymal transition. (predicted)	Rao et al., 2020
hsa-miR-30c-5p	MIMAT0000244	Directly targets MAPK1 to regulate proliferation and migration. Negatively regulates protein secretion. (validated)	Rao et al., 2020
hsa-miR-96-5p	MIMAT0000095	Inhibits apoptosis by targeting caspase-9 gene. (validated)	Rao et al., 2020
hsa-miR-142-5p	MIMAT0000433	Acts as a negative regulator in TGF-β pathway by targeting SMAD3 and suppresses TGF-β-induced growth inhibition. (validated)	Rao et al., 2020
hsa-miR-9-5p	MIMAT0000441	Plays a role in growth, invasion, migration, and epithelial–mesenchymal transition. Negatively regulates cell adhesion and janus kinase–signal transducer and activator of transcription(JAK/STAT) pathway. (validated)	Rao et al., 2020
hsa-miR-143-3p	MIMAT0000435	It regulates proliferation, migration, and invasion. Negativelyregulates angiogenesis, actin cytoskeleton organization, regulation of blood vessel and endothelial cell proliferation. (validated)	Rao et al., 2020
hsa-miR-302a-3p	MIMAT0000684	Post-transcriptional gene silencing. Overexpression can inhibit proliferation and promote apoptosis. May modulate epithelial–mesenchymal transition. (validated)	Rao et al., 2020
hsa-miR-223-3p	MIMAT00003280	Plays a protective role against endothelial injury, inhibits cell proliferation and migration, and negatively regulates inflammatory response and necrotic cell death. (validated)	Rao et al., 2020
hsa-miR-630	MIMAT0003299	Involved in the regulation of apoptosis. (predicted)	Drewryet al., 2018
hsa-miR-451a	MIMAT0001631	Associated with cell proliferation, migration, and apoptosis through targeting activating transcription factor 2 (ATF2) signaling pathway. It can inhibit hepatic gluconeogenesis and alleviate hyperglycemia. (predicted)	Drewryet al., 2018
has-miR-637	MIMAT0003307	Involved in tryosine metabolism and endocytosis. (predicted)	Hindle, 2019
hsa-miR-4725-3p	MIMAT0019844	Involved in MAPK signaling pathway, p53 signaling pathway, and regulation of actin cytoskeleton. (predicted)	Hindle, 2019
hsa-miR-433-3p	MIMAT0001627	Involved in MAPK signaling pathway, p53 signaling pathway, FOXO signaling pathway, and regulation of actin cytoskeleton. (predicted)	Hindle, 2019
hsa-miR-302d-3p	MIMAT0000718	Involved in tryosine metabolism and endocytosis. (predicted)	Drewry et al., 2018

## Data Availability

Not applicable.

## References

[1] Ritch R. (2008). The management of exfoliative glaucoma. Prog. Brain Res..

[2] Ritch R., Schlotzer-Schrehardt U., Konstas A.G. (2003). Why is glaucoma associated with exfoliation syndrome?. Progr. Retin. Eye Res..

[3] Lee R.K. (2008). The molecular pathophysiology of exfoliation glaucoma. Curr. Opin. Ophthalmol..

[4] Jeng S.M., Karger R.A., Hodge D.O., Burke J.P., Johnson D.H., Good M.S. (2007). The risk of glaucoma in exfoliation syndrome. J. Glaucoma.

[5] Thorleifsson G., Magnusson K.P., Sulem P., Walters G., Gudbjartsson D.F., Stefansson H., Jonsson T., Jonasdottir A., Jonasdottir A., Stefansdottir G. (2007). Common sequence variants in the LOXL1 gene confer susceptibility to exfoliation glaucoma. Science.

[6] Aung T., Ozaki M., Lee M.C., Schlötzer-Schrehardt U., Thorleifsson G., Mizoguchi T., Igo R.P., Haripriya A., Williams S.E., Astakhov Y.S. (2017). Genetic association study of exfoliation syndrome identifies a protective rare variant at LOXL1 and five new susceptibility loci. Nat. Genet..

[7] Aung T., Ozaki M., Mizoguchi T., Allingham R.R., Li Z., Haripriya A., Nakano S., Uebe S., Harder J.M., Chan A.S. (2015). A common variant mapping to CACNA1A is associated with susceptibility to exfoliation syndrome. Nat. Genet..

[8] Dewundara S., Pasquale L.R. (2015). Exfoliation syndrome: A disease with an environmental component. Curr. Opin. Ophthalmol..

[9] Ghaffari S.M., Damji K.F., Unsworth L.D. (2020). Recent advances in risk factors associated with ocular exfoliation syndrome. Acta Ophthalmol..

[10] Rao A., Chakraborty M., Roy A., Sahay P., Pradhan A., Raj N. (2020). Differential miRNA Expression: Signature for Glaucoma in Exfoliation. Clin. Ophthalmol..

[11] Filipowicz W., Bhattacharyya S.N., Sonenberg N. (2008). Mechanisms of post-transcriptional regulation by microRNAs: Are the answers in sight?. Nat. Rev. Genet..

[12] Chen K., Rajewsky N. (2007). The evolution of gene regulation by transcription factors and microRNAs. Nat. Rev. Genet..

[13] Bentwich I., Avniel A., Karov Y., Aharonov R., Gilad S., Barad O., Barzilai A., Einat P., Einav U., Meiri E. (2005). Identification of hundreds of conserved and nonconserved human microRNAs. Nat. Genet..

[14] Nitschke L., Tewari A., Coffin S.L., Xhako E., Pang K., Gennarino V.A., Johnson J.L., Blanco F.A., Liu Z., Zoghbi H.Y. (2020). miR760 regulates ATXN1 levels via interaction with its 5′ untranslated region. Genes Dev..

[15] Guo R., Shen W., Su C., Jiang S., Wang J. (2017). Relationship between the pathogenesis of glaucoma and miRNA. Ophthalmic Res..

[16] Undi R.B., Kandi R., Gutti R.K. (2013). MicroRNAs as Haematopoiesis Regulators. Adv. Hematol..

[17] Lu B., Christensen I.T., Ma L.W., Wang X.L., Jiang L.F., Wang C.X., Feng L., Zhang J.S., Yan Q.C. (2018). miR-24-p53 pathway evoked by oxidative stress promotes lens epithelial cell apoptosis in age-related cataracts. Mol. Med. Rep..

[18] Wang Y., Li F., Wang S. (2016). MicroRNA-93 is overexpressed and induces apoptosis in glaucoma trabecular meshwork cells. Mol. Med. Rep..

[19] Jayaram H., Cepurna W.O., Johnson E.C., Morrison J.C. (2015). MicroRNA Expression in the Glaucomatous Retina. Investig. Ophthalmol. Vis. Sci..

[20] Weber J.A., Baxter D.H., Zhang S., Huang D.Y., How Huang K., Jen L.M., Galas D.J., Wang K. (2010). The microRNA spectrum in 12 body fluids. Clin. Chem..

[21] Tanaka Y., Tsuda S., Kunikata H., Sato J., Kokubun T., Yasuda M., Nishiguchi K.M., Inada T., Nakazawa T. (2014). Profiles of extracellular miRNAs in the aqueous humor of glaucoma patients assessed with a microarray system. Sci. Rep..

[22] Lee Y.H., Kim S.Y., Bae Y.S. (2014). Upregulation of miR-760 and miR-186 is associated with replicative senescence in human lung fibroblast cells. Mol. Cells.

[23] Liu Y., Chen Y., Wang Y., Zhang X., Gao K., Chen S., Zhang X. (2018). microRNA profiling in glaucoma eyes with varying degrees of optic neuropathy by using next-generation sequencing. Investig. Ophthalmol. Vis. Sci..

[24] Peng C.H., Liu J.H., Woung L.C., Lin T.J., Chiou S.H., Tseng P.C., Du W.Y., Cheng C.K., Hu C.C., Chien K.H. (2012). MicroRNAs and cataracts: Cor-relation among let-7 expression, age and severity of lens opacity. Br. J. Ophthalmol..

[25] Sand M., Gambichler T., Sand D., Skrygan M., Altmeyer P., Bechara F.G. (2009). MicroRNAs and the skin: Tiny players in the body’s largest organ. J. Dermatol. Sci..

[26] Khee S.G., Yusof Y.A., Makpol S. (2014). Expression of senescence-associated microRNAs and target genes in cellular aging and modulation by tocotrienol-rich fraction. Oxidative Med. Cell. Longev..

[27] Kim J., Yoon H., Chung D.E., Brown J.L., Belmonte K.C., Kim J. (2016). miR-186 is decreased in aged brain and suppresses BACE 1 expression. J. Neurochem..

[28] Lou G., Xu W., Dong F., Chen G., Liu Y. (2018). Plasma miR-17, miR-20a, miR-20b and miR-122 as potential biomarkers for diagnosis of NAFLD in type 2 diabetes mellitus patients. Life Sci..

[29] Xiu C., Jiang J., Song R. (2020). Expression of miR-34a in cataract rats and its related mechanism. Exp. Ther. Med..

[30] Pan Y.J., Wei L.L., Wu X.J., Huo F.C., Mou J., Pei D.S. (2017). MiR-106a-5p inhibits the cell migration and invasion of renal cell carcinoma through targeting PAK5. Cell Death Dis..

[31] Que T., Song Y., Liu Z., Zheng S., Long H., Li Z., Liu Y., Wang G., Liu Y., Zhou J. (2015). Decreased miRNA-637 is an unfavorable prognosis marker and promotes glioma cell growth; migration and invasion via direct targeting Akt1. Oncogene.

[32] Shi X.B., Xue L., Yang J., Ma A.H., Zhao J., Xu M., Tepper C.G., Evans C.P., Kung H.J., deVere White R.W. (2016). An androgen-regulated miRNA suppresses Bak1 expression and induces BACE1 expression. J. Neurochem..

[33] Ye D., Zhang T. (2007). Androgen-independent growth of pros-tate cancer cells. Proc. Natl. Acad. Sci. USA.

[34] Thomson J.M., Newman M., Parker J.S., Morin-Kensicki E.M., Wright T., Hammond S.M. (2006). Extensive post-transcriptional regulation of microRNAs and its implications for cancer. Genes Dev..

[35] Xu J., Li J., Zheng T.H., Bai L., Liu Z.J. (2016). MicroRNAs in the occurrence and development of primary hepatocellular carcinoma. Adv. Clin. Exp. Med..

[36] Zhang J.F., He M.L., Fu W.M., Wang H., Chen L.Z., Zhu X., Chen Y., Xie D., Lai P., Chen G. (2011). Primate-specific microRNA-637 inhibits tumorigenesis in hepatocellular carcinoma by disrupting signal transducer and activator of transcription 3 signaling. Hepatology.

[37] Zhang J.F., Fu W.M., He M.L., Wang H., Wang W.M., Yu S.C., Bian X.W., Zhou J., Lin M.C., Lu G. (2011). MiR-637 maintains the balance between adipocytes and osteoblasts by directly targeting Osterix. Mol. Biol. Cell.

[38] Youngblood H., Cai J., Drewry M.D., Helwa I., Hu E., Liu S., Yu H., Mu H., Hu Y., Perkumas K. (2020). Expression of mRNAs; miRNAs; and lncRNAs in Human Trabecular Meshwork Cells Upon Mechanical Stretch. Investig. Ophthalmol. Vis. Sci..

[39] Jayaram H., Phillips J.I., Lozano D.C., Choe T.E., Cepurna W.O., Johnson E.C., Morrison J.C., Gattey D.M., Saugstad J.A., Keller K.E. (2017). Comparison of MicroRNA expression in aqueous humor of normal and primary open-angle glaucoma patients using PCR arrays: A pilot study. Investig. Ophthalmol. Vis. Sci..

[40] Li X., Zhao F., Xin M., Li G., Luna C., Li G., Zhou Q., He Y., Yu B., Olson E. (2017). Regulation of intraocular pressure by microRNA cluster miR-143/145. Sci. Rep..

[41] Drewry M.D., Challa P., Kuchtey J.G., Navarro I., Helwa I., Hu Y., Mu H., Stamer W.D., Kuchtey R.W., Liu Y. (2018). Differentially expressed microRNAs in the aqueous humor of patients with exfoliation glaucoma or primary open-angle glaucoma. Hum. Mol. Genet..

[42] Kosior-Jarecka E., Czop M., Gasińska K., Wróbel-Dudzińska D., Zalewski D.P., Bogucka-Kocka A., Kocki J., Żarnowski T. (2021). MicroRNAs in the aqueous humor of patients with different types of glaucoma. Graefe’s Arch. Clin. Exp. Ophthalmol..

[43] Hindle A.G., Thoonen R., Jasien J.V., Grange R.M.H., Amin K., Wise J., Ozaki M., Ritch R., Malhotra R., Buys E.S. (2019). Identification of Candidate miRNA Biomarkers for Glaucoma. Investig. Ophthalmol. Vis. Sci..

[44] Tomczyk-Socha M., Baczyńska D., Przeździecka-Dołyk J., Turno-Kręcicka A. (2020). MicroRNA-125b overexpression in exfoliation syndrome. Adv. Clin. Exp. Med. Off. Organ Wroc. Med. Univ..

[45] Moschos M.M., Dettoraki M., Karekla A., Lamprinakis I., Damaskos C., Gouliopoulos N., Tibilis M., Gazouli M. (2020). Polymorphism analysis of miR182 and CDKN2B genes in Greek patients with primary open angle glaucoma. PLoS ONE.

[46] He J., Zhao J., Zhu W., Qi D., Wang L., Sun J., Wang B., Ma X., Dai Q., Yu X. (2016). MicroRNA biogenesis pathway genes polymorphisms and cancer risk: A systematic review and meta-analysis. PeerJ.

[47] Chatzikyriakidou A., Founti P., Melidou A., Minti F., Bouras E., Anastasopoulos E., Pappas T., Haidich A.B., Lambropoulos A., Topouzis F. (2018). MicroRNA-related polymorphisms in exfoliation syndrome; pseudoexfoliative glaucoma; and primary open-angle glaucoma. Ophthalmic Genet..

[48] Saeki M., Watanabe M., Inoue N., Tokiyoshi E., Takuse Y., Arakawa Y., Hidaka Y., Iwatani Y. (2016). DICER and DROSHA gene expression and polymorphisms in autoimmune thyroid diseases. Autoimmunity.

[49] Luna C., Li G., Qiu J., Epstein D.L., Gonzalez P. (2009). Role of miR-29b on the regulation of the extracellular matrix in human trabecular meshwork cells under chronic oxidative stress. Mol. Vis..

[50] Chakraborty M., Sahay P., Rao A. (2021). Primary Human Trabecular Meshwork Model for Pseudoexfoliation. Cells.

[51] Sharma S., Chataway T., Klebe S., Griggs K., Martin S., Chegeni N., Dave A., Zhou T., Ronci M., Voelcker N.H. (2018). Novel protein constituents of pathological ocular exfoliation syndrome deposits identified with mass spectrometry. Mol. Vis..

[52] Wolosin J.M., Ritch R., Bernstein A.M. (2018). Is Autophagy Dysfunction a Key to Exfoliation Glaucoma?. J. Glaucoma.

[53] Rao A., Sahay P., Chakraborty M., Prusty B.K., Srinivasan S., Jhingan G.D., Mishra P., Modak R., Suar M. (2021). Switch to Autophagy the Key Mechanism for Trabecular Meshwork Death in Severe Glaucoma. Clin. Ophthalmol..

[54] De Juan-Marcos L., Escudero-Domínguez F.A., Hernández-Galilea E., Cruz-González F., Follana-Neira I., González-Sarmiento R. (2018). Investigation of Association between Autophagy-Related Gene Polymorphisms and Exfoliation Syndrome and Exfoliation Glaucoma in a Spanish Population. Semin. Ophthalmol..

[55] Want A., Gillespie S.R., Wang Z., Gordon R., Iomini C., Ritch R., Wolosin J.M., Bernstein A.M. (2016). Autophagy and Mitochondrial Dysfunction in Tenon Fibroblasts from Exfoliation Glaucoma Patients. PLoS ONE.

[56] Chistiakov D.A., Sobenin I.A., Revin V.V., Orekhov A.N., Bobryshev Y.V. (2014). Mitochondrial aging and age-related dysfunction of mitochondria. BioMed Res. Int..

[57] Manickam A.H., Michael M.J., Ramasamy S. (2017). Mitochondrial genetics and therapeutic overview of Leber’s hereditary optic neuropathy. Indian J. Ophthalmol..

[58] Tanwar M., Dada T., Sihota R., Dada R. (2010). Mitochondrial DNA analysis in primary congenital glaucoma. Mol. Vis..

[59] Izzotti A., Longobardi M., Cartiglia C., Sacca S.C. (2011). Mitochondrial damage in the trabecular meshwork occurs only in primary open-angle glaucoma and in pseudoexfoliative glaucoma. PLoS ONE.

[60] Porter K., Nallathambi J., Lin Y., Liton P.B. (2013). Lysosomal basification and decreased autophagic flux in oxidatively stressed trabecular meshwork cells: Implications for glaucoma pathogenesis. Autophagy.

[61] Liton P.B., Lin Y., Gonzalez P., Epstein D.L. (2009). Potential role of lysosomal dysfunction in the pathogenesis of primary open angle glaucoma. Autophagy.

[62] Abu-Amero K.K., Morales J., Bosley T.M. (2006). Mitochondrial abnormalities in patients with primary open-angle glaucoma. Investig. Ophthalmol. Vis. Sci..

[63] Sundaresan P., Simpson D.A., Sambare C., Duffy S., Lechner J., Dastane A., Dervan E.W., Vallabh N., Chelerkar V., Deshpande M. (2015). Whole-mitochondrial genome sequencing in primary open-angle glaucoma using massively parallel sequencing identifies novel and known pathogenic variants. Genet. Med. Off. J. Am. Coll. Med. Genet..

[64] Abu-Amero K.K., Bosley T.M., Morales J. (2008). Analysis of nuclear and mitochondrial genes in patients with exfoliation glaucoma. Mol. Vis..

[65] Sirohi K., Swarup G. (2016). Defects in autophagy caused by glaucoma-associated mutations in optineurin. Exp. Eye Res..

[66] Bosley T.M., Hellani A., Spaeth G.L., Myers J., Katz L.J., Moster M.R., Milcarek B., Abu-Amero K.K. (2011). Down-regulation of OPA1 in patients with primary open angle glaucoma. Mol. Vis..

[67] Davies V., Votruba M. (2006). Focus on molecules: The OPA1 protein. Exp. Eye Res..

[68] Lenaers G., Reynier P., Elachouri G., Soukkarieh C., Olichon A., Belenguer P., Baricault L., Ducommun B., Hamel C., Delettre C. (2009). OPA1 functions in mitochondria and dysfunctions in optic nerve. Int. J. Biochem. Cell Biol..

[69] Singh L.N., Crowston J.G., Lopez Sanchez M.I.G., Van Bergen N.J., Kearns L.S., Hewitt A.W., Yazar S., Mackey D.A., Wallace D.C., Trounce I.A. (2018). Mitochondrial DNA Variation and Disease Susceptibility in Primary Open-Angle Glaucoma. Investig. Ophthalmol. Vis. Sci..

[70] Coca-Prados M. (2014). The blood-aqueous barrier in health and disease. J. Glaucoma.

[71] Bill A. (1986). The blood-aqueous barrier. Trans. Ophthalmol. Soc. UK.

[72] Küchle M., Vinores S.A., Mahlow J., Green W.R. (1996). Blood-aqueous barrier in exfoliation syndrome: Evaluation by immunohistochemical staining of endogenous albumin. Graefe’s Arch. Clin. Exp. Ophthalmol..

[73] Wiggs J.L., Pawlyk B., Connolly E., Adamian M., Miller J.W., Pasquale L.R., Haddadin R.I., Grosskreutz C.L., Rhee D.J., Li T. (2014). Disruption of the blood-aqueous barrier and lens abnormalities in mice lacking lysyl oxidase-like 1 (LOXL1). Investig. Ophthalmol. Vis. Sci..

[74] Küchle M., Nguyen N.X., Hannappel E., Naumann G.O. (1995). The blood-aqueous barrier in eyes with exfoliation syndrome. Ophthalmic Res..

[75] Yavrum F., Elgin U., Kocer Z.A., Fidanci V., Sen E. (2021). Evaluation of aqueous humor and serum clusterin levels in patients with glaucoma. BMC Ophthalmol..

[76] Doudevski I., Rostagno A., Cowman M., Liebmann J., Ritch R., Ghiso J. (2014). Clusterin and complement activation in exfoliation glaucoma. Investig. Ophthalmol. Vis. Sci..

[77] Zenkel M., Kruse F.E., Jünemann A.G., Naumann G.O., Schlötzer-Schrehardt U. (2006). Clusterin deficiency in eyes with exfoliation syndrome may be implicated in the aggregation and deposition of pseudoexfoliative material. Investig. Ophthalmol. Vis. Sci..

[78] Yildirim Z., Yildirim F., Uçgun N.I., Sepici-Dinçel A. (2013). The role of the cytokines in the pathogenesis of exfoliation syndrome. Int. J. Ophthalmol..

[79] Kondkar A., Azad T.A., Almobarak F., Kalantan H., Al-Obeidan S.A., Abu-Amero K.K. (2018). Elevated levels of plasma tumor necrosis factor alpha in patients with exfoliation glaucoma. Clin. Ophthalmol..

[80] Eraslan N., Elgin U., Şen E., Kilic A., Yilmazbas P. (2018). Comparison of total/active ghrelin levels in primary open angle glaucoma; exfoliation glaucoma and exfoliation syndrome. Int. J. Ophthalmol..

[81] Bleich S., Roedl J., Von Ahsen N., Schlötzer-Schrehardt U., Reulbach U., Beck G., Kruse F.E., Naumann G.O., Kornhuber J., Jünemann A.G. (2004). Elevated homocysteine levels in aqueous humor of patients with exfoliation glaucoma. Am. J. Ophthalmol..

[82] Kuroki M., Voest E.E., Amano S., Beerepoot L.V., Takashima S., Tolentino M., Kim R.Y., Rohan R.M., Colby K.A., Yeo K.T. (1996). Reactive oxygen intermediates increase vascular endothelial growth factor expression in vitro and in vivo. J. Clin. Investig..

[83] Borazan M., Karalezli A., Kucukerdonmez C., Bayraktar N., Kulaksizoglu S., Akman A., Akova Y.A. (2010). Aqueous humor and plasma levels of vascular endothelial growth factor and nitric oxide in patients with exfoliation syndrome and exfoliation glaucoma. J. Glaucoma.

[84] Aiello L.P., Northrup J.M., Keyt B.A., Takagi H., Iwamoto M.A. (1995). Hypoxic regulation of vascular endothelial growth factor in retinal cells. Arch. Ophthalmol..

